# Antibody responses against influenza A decline with successive years of annual influenza vaccination: results from an Australian Healthcare Worker cohort

**DOI:** 10.21203/rs.3.rs-4854923/v1

**Published:** 2024-09-16

**Authors:** Sheena Sullivan, Arseniy Khvorov, Louise Carolan, Leslie Dowson, Jessica Hadiprodjo, Stephany Sánchez-Ovando, Yi Liu, Vivian Leung, David Hodgson, Christopher Blyth, Marion Macnish, Allen Cheng, Michelle Hagenauer, Julia Clark, Sonia Dougherty, Kristine Macartney, Archana Koirala, Ameneh Khatami, Ajay Jadhav, Helen Marshall, Kathryn Riley, Peter Wark, Catherine Delahunty, Kanta Subbarao, Adam Kucharski, Annette Fox

**Affiliations:** Monash University; Peter Doherty Insitute for Infection and Immunity; Peter Doherty Insitute for Infection and Immunity; University of Melbourne; University of Melbourne; University of Melbourne; University of Melbourne; University of Melbourne; London School of Hygiene and Tropical Medicine; Telethon Kids Institute; Telethon Kids Institute; Alfred Health and Monash University; Alfred Health and Monash University; Queensland Children’s Hospital; Queensland Children’s Hospital; University of Sydney; The Children’s Hospital at Westmead; The Children’s Hospital at Westmead; The Children’s Hospital at Westmead; Adelaide Medical School and Robinson Research Institute; Adelaide Medical School and Robinson Research Institute; John Hunter Hospital; University of Newcastle; University of Melbourne; London School of Hygiene and Tropical Medicine; Peter Doherty Institute for Infection and Immunity

## Abstract

Influenza vaccine effectiveness and immunogenicity can be compromised with repeated vaccination. We assessed immunological markers in a cohort of healthcare workers (HCW) from six public hospitals around Australia during 2020–2021. Sera were collected pre-vaccination and ~14 and ~ 180 days post-vaccination and assessed in haemagglutination inhibition assay against egg-grown vaccine and equivalent cell-grown viruses. Responses to vaccination were compared by the number of prior vaccinations. Baseline sera were available for 595 HCW in 2020 and 1031 in 2021. 5% had not been vaccinated during five years prior to enrolment and 55% had been vaccinated every year. Post-vaccination titres for all vaccine antigens were lowest among HCW vaccinated in all 5-prior years and highest among HCW with 0 or 1 prior vaccinations, even after adjustment. This was observed for both influenza A subtypes and was dependent on pre-vaccination titre. Expanded cohorts are needed to better understand how this translates to vaccine effectiveness.

## Introduction

Influenza vaccines work by stimulating production of antibodies against the haemagglutinin protein–the principal protein responsible for virus infectivity [1]. Haemagglutinin is also the influenza protein that mutates most rapidly. As a result, influenza vaccines are reformulated annually to keep pace with virus evolution, and annual re-vaccination with the updated vaccines is recommended to ensure protection against contemporary viruses [2].

Although annual revaccination should represent our best option for protection against currently-circulating influenza viruses, it has long been known that repeated vaccination may, in fact, attenuate vaccine effectiveness. This was first reported in the 1970s [3], and has been re-visited on a number of occasions in vaccine effectiveness [4–6] and immunogenicity studies [7–12]. The effects have typically appeared to be worst for A(H3N2) viruses, which exhibits greater diversity than other human influenza viruses, making it challenging to identify a candidate vaccine virus (CVV) able to stimulate broad antibody coverage against circulating viruses [13]. However, A(H1N1)pdm09 has also begun to exhibit increasing diversity and attenuated effectiveness among repeat vaccinees has been observed for these viruses [4].

The effects are not consistent across seasons, which may be a consequence of varying antigenic distances between successive vaccine viruses and circulating viruses [5]. When the vaccine antigen is not updated, but predominant circulating viruses have antigenically drifted away from the CVV, the effects of repeated vaccination appear to be worst [14]. In such a scenario, vaccination may stimulate a focussed antibody response targeted at epitopes that have since been superseded and these antibodies are therefore incapable of neutralising the antigenically drifted viruses [15]. Conversely, positive interference may also occur when vaccine antigens are updated, stimulating both recall of prior antibodies as well as generation of new antibodies, the combination of which can provide broader protection against circulating viruses [11].

To better understand the mechanisms underlying observations of reduced immunogenicity and effectiveness in repeatedly-vaccinated persons, we established a multi-year cohort of healthcare workers (HCW) monitored for post-vaccination antibody responses and vaccine failures. HCW are recommended for priority influenza vaccination by the World Health Organization (WHO) [16] and are therefore a highly vaccinated group in many countries, including Australia. The cohort was established in April 2020, just as COVID-19 pandemic restrictions were implemented, which impeded recruitment. However, the first two years of the pandemic were also accompanied by local extinction of influenza [17]. This presented an opportunity to examine the post-vaccination antibody kinetic in a period during which infections were extremely rare. Here, we present the results from the first two years of this cohort.

## Methods

### Setting and participants

The cohort included healthcare workers (HCW) from six public health services around Australia: the Queensland Children’s Hospital, Brisbane; the John Hunter Hospital, Newcastle; the Children’s Hospital at Westmead, Sydney; Alfred Health, Melbourne; the Women and Children’s Hospital, Adelaide; and the Perth Children’s Hospital, Perth. These hospitals were chosen because they participate in patient influenza surveillance [18], and because they provided good geographic coverage, which was hoped to overcome potential variations in seasonality and predominant virus circulation [19].

The cohort was an open cohort, permitting new recruitment each year (2020–2023) to reach the target sample size of 250 HCW per site. HCW were eligible to participate if they were employees, students or volunteers at participating health services, were aged 18–64 years with no known contraindications to influenza vaccines and had not yet received the influenza vaccine in their enrolment year. Enrolment commenced on 2 April 2020. HCWs were asked to complete a brief questionnaire to collect demographic information, employment category, medical history and 5-year influenza vaccination history. Blood was collected by venepuncture from participants immediately prior to vaccination (pre-vaccination), 14–21 days post-vaccination and around the end of the usual Australian influenza season (October-November).

### Ethical considerations

This study was approved by the Human Research Ethics Committee of the Royal Melbourne Hospital (HREC Reference Number: HREC/54245/MH-2019). All study staff were trained in Good Clinical Practice and Human Subjects Protection. Written informed consent was obtained from all HCWs upon enrolment.

### Vaccines

At all sites, state governments provide influenza vaccine to all public hospitals for staff vaccination. At the time of the study, influenza vaccination was not mandatory for HCW. However, there was a strong influenza campaign in 2020 to avoid the risk of a dual influenza and SARS-CoV-2 epidemic. The state of Victoria set very high targets for vaccine coverage among its HCWs (>90%) [20], and New South Wales also introduced vaccination mandates for certain clinical staff in 2019 [21]. Given the strong recommendation for vaccination among HCW, it was expected that a majority of HCW willing to participate would have received multiple prior vaccinations. Therefore, extra effort was made to recruit vaccine-naive participants with a target of at least 10 per site per year.

In 2020, HCW received quadrivalent influenza vaccines containing egg-grown inactivated viruses that were A/Brisbane/02/2018 (H1N1pdm09)-like, A/South Australia/34/2019 (H3N2)-like, B/Washington/02/2019 (B/Victoria)-like virus and B/Phuket/3073/2013 (B/Yamagata)-like virus. In 2021, the formulation was updated to include 2 new influenza A viruses, an A/Victoria/2570/2019 (H1N1pdm09)-like virus and an A/Hong Kong/2671/2019 (H3N2)-like virus. Only responses against the influenza A antigens are reported here.

### Serological assays

Sera were tested for the presence of antibodies against each of the vaccine influenza A antigens using the haemagglutination inhibition (HI) assay as previously described [22]. Both egg- and cell-grown influenza antigens were used, where the egg-grown antigen provides an indication of response to the vaccine, while cell-grown antigen provides an indication of the level of protection a person might have against circulating viruses. Sera were treated with receptor destroying enzyme (Denka Sieken) to remove non-specific haemagglutination inhibitors and were adsorbed with a mixture of erythrocytes from turkeys (H1N1pdm09) and guinea pigs (H3N2) to remove non-specific haemagglutination. Sera were diluted 2-fold starting at 1:10 to a maximum dilution of 1:10240. HI antibody titres were read using a CypherOne automated reader (InDevR, Colorado, USA) as the reciprocal of the highest serum dilution causing complete inhibition of agglutination.

### Statistical analysis

Where relevant, HI titres of <10 were imputed as 5. HI titres of 10240 could potentially be >10240 but were not imputed. Titres were log_2_ transformed for analyses, and later back-transformed to titre values for interpretation. We initially assessed crude geometric mean titres (GMT), seroconversion (proportion exhibiting at least a 4-fold rise in titre) and seropositivity (proportion with titres >=40), by prior vaccination status. The association between post-vaccination GMT and the number of prior vaccinations was assessed by linear regression and the Jonckheere-Terpstra test for trend.

GMTs and geometric mean ratios (GMRs) were also estimated using a log-linear regression model where the outcome was the day 14 log-post-vaccination titre or geometric mean fold rise. In univariable models, prior vaccination status was modelled as a linear term to estimate the incremental effect of each successive prior vaccination. In multivariable analysis, prior vaccination was modelled as an ordinal term to allow for non-monotonicity. Potential covariates that were explored included pre-vaccination titre (centred at a titre of 5), age (centred to 18 years), sex, body mass index (BMI), the presence of any health conditions and vaccine brand. The full model including all covariates was compared with more parsimonious models based on Akaike Information Criterion (AIC). For each post-vaccination outcome explored, the same model parameters were used for each virus examined. Predicted GMTs and GMRs were plotted for visual assessment and compared with crude (observed) values. The same approach was used to predict seroconversion and seropositivity in melogistic regression models. All statistical analyses were performed using R version 4.2.2 (R Foundation for Statistical Computing, Vienna, Austria).

### Data availability

The datasets used and/or analysed during the current study available from the corresponding author on reasonable request.

### Code availability

The underlying R scripts used for this study are not publicly available but may be made available to qualified researchers on reasonable request from the corresponding author.

### Ethics approval

This study was performed in line with the principles of the Declaration of Helsinki. The study protocol and protocol addendums for follow-up of COVID-19 vaccinations and SARS-CoV-2 infections were approved by The Royal Melbourne Hospital Human Research Ethics Committee (HREC/54245/MH-2019).

### Consent to participate

Written informed consent was obtained from all individuals who participated in the study.

## Results

### Baseline characteristics

**Figure 1:**
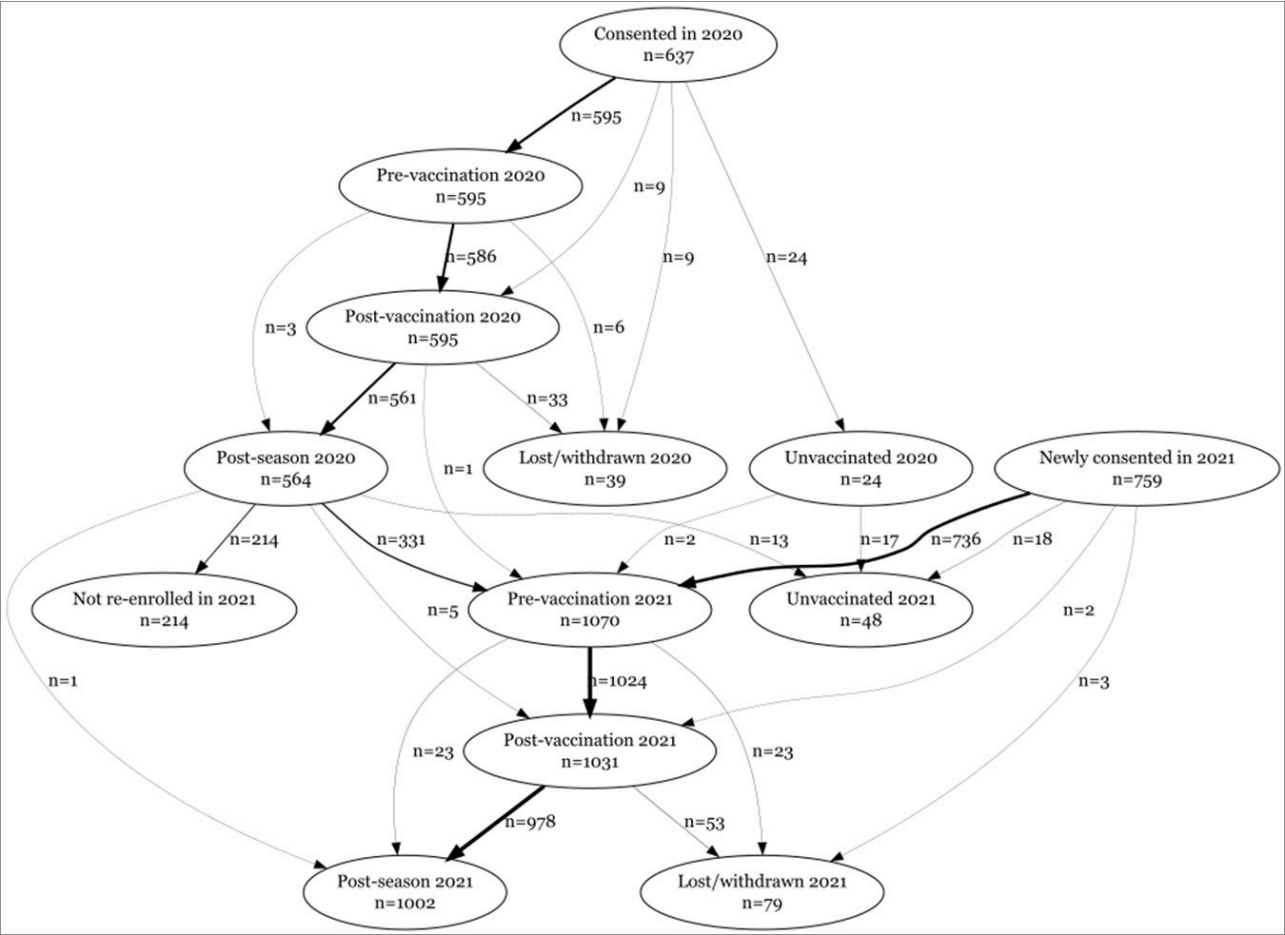
Strobe flowchart showing participants enrolled in the cohort for whom samples were available for serology at pre-vaccination, post-vaccination and post-season visits.

In 2020, 637 HCW were recruited across 6 sites, of whom 24 opted not to be vaccinated in 2020 and were therefore not considered in this analysis. Pre-vaccination and post-vaccination blood samples were available for 595 vaccinated HCWs (586 with both visits), and end-of-season blood samples were available for 564. The median time between vaccination and post-vaccination blood draw was 15 days (IQR: 14, 18) and the median time between vaccination and end-of-year blood draw was 174 days (IQR: 165, 188) (Supplementary Figure 1). In 2021, the number newly recruited was 759, while 339 recruited in 2020 (including 2 who were unvaccinated in 2020), were vaccinated in 2021 and continued follow up. Eighteen of the HCWs newly recruited in 2021 were unvaccinated and not considered further. Pre-vaccination blood samples were available for 1070 vaccinated HCWs, post-vaccination samples for 1031 and end-of-season samples for 1002. The median time between vaccination and post-vaccination blood draw was 15 days (IQR: 14, 19) and the median time between vaccination and end-of-year blood draw was 165 days (QR: 154,184). See Figure 1 for the STROBE flowchart detailing patient recruitment and follow-up and Supplementary Figure 1 for a summary of follow-up times.

Demographic and workplace characteristics are presented in Table 1 by study year. HCW were a median of 39 years at recruitment (39y in 2020; 40y in 2021) and predominantly female (81% in 2020; 85% in 2021). In both study years, most HCW were full-time employed (57% in 2020; 62% in 2021) and around half were in clinical roles (53% in 2020; 46% in 2021). Fourteen percent had at least one high risk condition (13% in 2020; 15% in 2021).

### A(H1N1)pdm09 antibody titres over the course of vaccination

The A(H1N1)pdm09 vaccine antigens included in egg-based vaccines in 2020 and 2021 were from genetically distinct subgroups, with A/Brisbane/02/2018 in 6B.1A.1 subgroup, and A/Victoria/2570/2019 in the 6B.1A.5a.2 subgroup. Vaccine antigens for the 5 years prior to 2020 were also genetically distinct, being an A/Michigan/45/2015 6B.1 virus in 2017–19 and A/California/7/2009 in 2015–16 (Supplementary Figure 2).

**Figure 2:**
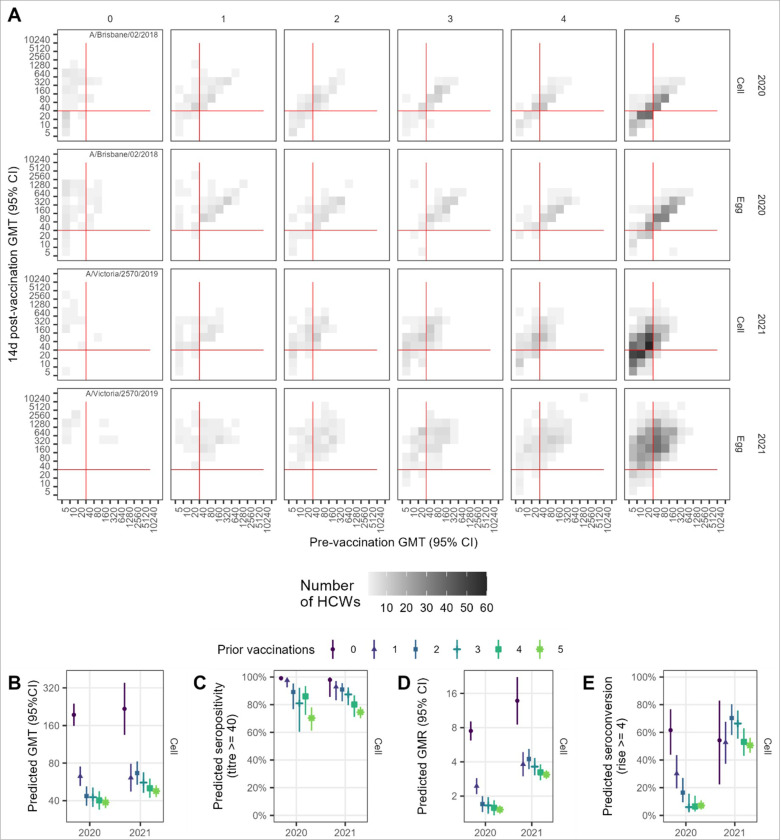
Vaccination induced HI antibody responses for A(H1N1)pdm09 by number of prior years’ vaccinated. A. Observed post vaccination geometric mean titres (GMTs) by pre-vaccination GMT for both cell and egg-grown antigens. Data convergence on the diagonal is indicative of minimal titre rise. Red lines indicate the seropositivity threshold titre of 40. B. Predicted GMT 14 days post-vaccination from linear regression model adjusting for baseline titre, vaccine brand, age, sex, and presence of any health conditions. C. Predicted seropositivity 14d post-vaccination for cell-grown antigen from the logistic regression model adjusting for baseline titre. D. Predicted geometric mean titre ratios (GMR) 14d post-vaccination from the linear regression model adjusting for baseline titre, vaccine brand, age. E. Predicted proportion of HCW who seroconverted 14d post-vaccination from the logistic regression model adjusting for baseline titre, vaccine brand, age. Panels D-E show results for cell-grown antigens only.

In both 2020 and 2021, pre-vaccination GMTs against both cell and egg-grown antigens were lowest among HCWs with 0-prior vaccinations at around 10–20, with the group with 5+ prior vaccinations having the next lowest pre-vaccination GMTs (Supplementary Table 1, Supplementary Figure 3A). In contrast, day 14 post-vaccination GMTs were higher in the 0-prior group compared with the 5-prior group. There was also a statistically significant trend towards increased post-vaccination titres with lower number of prior vaccinations in both 2020 and 2021, with an average decrease of at least 0.79 with each additional prior vaccination (Table 2). Post-vaccination titres were also dependent on the pre-vaccination titre (Figure 2A; Supplementary Figure 4A). Predicted post-vaccination GMTs from the model were adjusted for pre-vaccination titre (centred at 5), vaccine brand, age in decades (centred at 18 years), sex, BMI, and presence of any pre-existing health conditions and continued to show an inverse association with both number of prior vaccinations as well as a positive association with pre-vaccination titre (Figure 2B; Supplementary Table 2). Age and vaccine brand were also important predictors against egg-, but not cell-grown, antigens (Supplementary Table 2).

Pre-vaccination seropositivity was lowest for the 0-prior vaccination group, but those with 5-priorvaccinations had the next lowest seropositivity and this group remained lowest post-vaccination (Supplementary Table 1). For all vaccination groups seropositivity increased to above 50% post-vaccination for cell-grown antigens and was even higher for egg-grown antigens (above 85%), and seropositivity was sustained above pre-vaccination levels 6 months post-vaccination. There was a clear trend of decreasing seropositivity with increasing numbers of prior vaccinations in 2021 but not 2020 (Supplementary Figure 3B); however, a clearer trend emerged after adjustment for pre-vaccination titre (Figure 2B; Supplementary Figure 6).

Post-vaccination geometric mean titre rises (GMRs) were highest for the vaccine-naive group but were not very different among the 4 vaccine-experienced groups, all with a mean GMR of ~2 in 2020 and ranging from 3–4 against cell antigens and from 6–8 against egg antigens in 2021 (Supplementary Figure 3C; Supplementary Table 1). GMRs decreased with increasing pre-vaccination titre and fell below 4-fold for all prior vaccination groups with pre-vaccination titres exceeding 80 (Supplementary Figure 4C). Effects of prior vaccination remained substantial after adjusting for pre-vaccination titre, age at enrolment and vaccine brand (Supplementary Table 4, Supplementary Figure 7). Similarly, seroconversion was higher among the vaccine-naive, but not very different among vaccine-experienced groups (Supplementary Table 1; Supplementary Figure 3D). Adjustment for pre-vaccination titre, age and brand reduced the differences in seroconverted proportions between the vaccine-naive and vaccine-experienced, and, in 2021, revealed no apparent trend of declining seroconversion from 0 to 5-prior vaccinations (Supplementary Table 5, Supplementary Figure 8).

### A(H3N2) antibody titres over the course of vaccination

The vaccine administered in 2020 contained an A/South Australia/34/2019-like virus, which fell in the 3C.2a1b.2 genetic subgroup (Supplementary Figure 9). This virus was genetically distinct from the 2021 vaccine virus, A/Hong Kong/2671/2019, which fell in the 3C.2a1b.1b subgroup, and both were distinct from the vaccine viruses used in the 5 years prior to 2020. However, there were some shared epitopes, including the T160K substitution in the egg antigens of 2016–2021 vaccine strains, which is a known egg-acquired adaptation associated with a loss of glycosylation [23]. All vaccine strains apart from A/Hong Kong /2671/2019 contained several other glycosylation sites within antigenic sites A and B that were retained in egg-grown strains (Supplementary Table 6).

**Figure 3:**
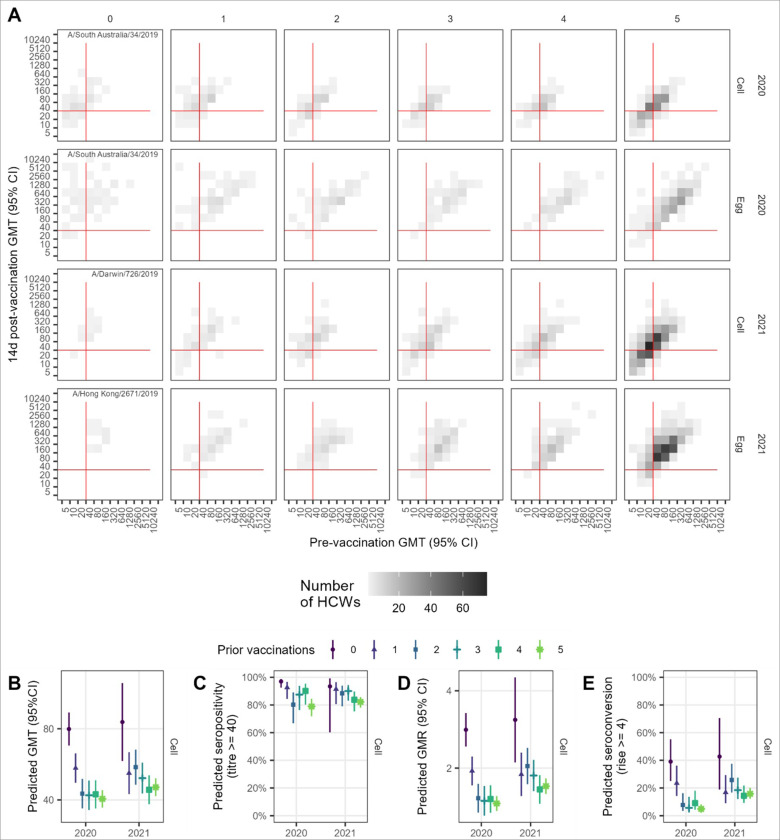
Vaccination induced HI antibody responses for A(H3N2) by number of prior years’ vaccinated. A.Observed post vaccination geometric mean titres (GMTs) by pre-vaccination GMT for both cell and egg-grown antigens. Data convergence on the diagonal is indicative of minimal titre rise. Red lines indicate the seropositivity threshold titre of 40. B. Predicted GMT 14 days post-vaccination from linear regression model adjusting for baseline titre, vaccine brand, age, sex, and presence of any health conditions. C. Predicted seropositivity 14d post-vaccination for cell-grown antigen from the logistic regression model adjusting for baseline titre. D. Predicted geometric mean titre ratios (GMR) 14d post-vaccination from the linear regression model adjusting for baseline titre, vaccine brand, age. E. Predicted proportion of HCW who seroconverted 14d post-vaccination from the logistic regression model adjusting for baseline titre, vaccine brand, age. Panels D-E show results for cell-grown antigens only.

As with A(H1N1)pdm09, responses to A(H3N2) antigens exhibited a pattern of declining GMTs by number of prior vaccinations (Supplementary Table 7; Supplementary Figure 10), and increasing GMTs with higher pre-vaccination titres (Figure 3A; Supplementary Figure 11). The trend was more apparent in 2021 than in 2020, with an expected reduction in GMT of 0.93 in 2020 and 0.87 in 2021 (Table 2), which was maintained after adjustment for pre-vaccination titre, vaccine brand, age, sex, BMI and pre-existing health conditions (Figure 3B; Supplementary Table 8, Supplementary Figure 12).

Post-vaccination seropositivity was high and above 65% for cell-grown antigens and above 90% for egg-grown antigens, consistent with higher post-vaccination HI titres against egg compared with cell-grown antigens (Supplementary Table 7; Supplementary Figure 10B). In 2020, the raw data suggested increasing seropositivity from 0 to 4 prior vaccinations; however, after adjustment for pre-vaccination titre, this trend reversed, albeit not monotonically (Figure 3C; Supplementary Table 9, Supplementary Figure 13).

Post-vaccination GMRs were highest for the vaccine-naïve group with a mean rise of 3.3 against cell-grown antigens (compared to mean rises ranging from 1.4 to 2.3-fold for the vaccine-experienced groups (Supplementary Table 6; Supplementary Figure 10C). Correspondingly, around half the vaccine-naïve HCWs seroconverted to cell-grown antigen (56% in 2020 and 47% in 2021), but fewer than half of the vaccine-experienced groups seroconverted, with seroconversion as low as 7.7% for those receiving 3-prior vaccinations in 2020 (Supplementary Table 7; Supplementary Figure 10D). Seroconversions were higher against egg-compared with cell-grown antigens, and for HCWs with lower pre-vaccination titres (Supplementary Figure 11). GMR and seroconversion trends were maintained after adjustment for pre-vaccination titre, age and vaccine brand (Figure 3C-D; Supplementary Tables 10 & 11; Supplementary Figures 14 & 15).

## Discussion

We observed decreasing post-vaccination antibody titres with increasing numbers of prior vaccinations in a cohort of Australian HCWs vaccinated with southern hemisphere quadrivalent vaccines in 2020 and 2021. Trends were not monotonic but were statistically significant for both influenza A subtypes. The magnitude of response to vaccination was consistently highest for the group with 0-prior, while those with at least 5 prior vaccinations often had the weakest response. Pre-vaccination titres and seropositivity for the 5-prior group were second lowest to the 0-prior group, suggesting that attenuating effects of prior vaccination are incremental and sustained. These observations were consistent across antigens. When adjusted for pre-vaccination titre and other covariates, the magnitude of differences between the vaccination groups sometimes diminished, but the trend of decreasing post-vaccination responses with increasing numbers of prior vaccinations was generally preserved or became clearer. While studies that only consider prior year vaccination report conflicting effects on vaccine immunogenicity [8, 12] the results presented here confirm previous observations from our group [9, 10] and others [8, 12] that repeated influenza vaccination over multiple years attenuates immunogenicity.

Our study revealed that a range of factors other than prior vaccination influence antibody responses to influenza vaccination, which may explain why relationships with number of prior vaccinations are not monotonic. Most importantly, pre-vaccination titre strongly predicted post-vaccination responses consistent with studies elsewhere including studies of post-infection titre rise [24]. It is plausible that if titres are high pre-vaccination, it may be difficult to observe post-vaccination titre rises and seroconversions. This has been referred to as the ceiling effect [24–26]. To avoid false ceiling effects associated with the limit of detection, we used an extended titration series where the highest dilutionwas 1:10240. Only three titrations reached 10240; by comparison, most (>90%) post-vaccination titres against cell-grown antigens were 160 or below. The role of pre-existing immunity, including repeated vaccination, may modify ceiling effects, as previously suggested [27]. The negative effect of pre-vaccination antibody on antibody responses may also account for differences in effects of prior vaccination between subtypes. For example, the predicted reduction in GMT with each subsequent prior vaccination was greater for A(H1N1)pdm09 than for A(H3N2), and pre-vaccination titres were generally higher for the latter.

In 2020, the A/Brisbane/02/2018 virus was included in the vaccine for the first time, as an update to the previous A(H1N1)pdm09 CVV, A/Michigan/45/2015, which had been used for 3 years. The CVV was again updated in 2021 to A/Victoria/2570/2019, which was antigenically quite distinct from the Brisbane and Michigan viruses. This appears to have led to a marked improvement in the antibody boost from vaccination in 2021, with higher GMRs and proportion seroconverted in 2021 compared with 2020, even for the vaccine-experienced groups. Previous studies have identified age cohort effects of reduced vaccine effectiveness attributable to recall of epitopes shared with older A(H1N1) viruses in some age groups [28], which can be exacerbated by repeated vaccination [29]. Further work is underway to better understand whether antigen recall and birth cohort effects may underlie this observation.

A(H3N2) seropositivity was comparable with and sometimes higher than for A(H1N1)pdm09. This is somewhat at odds with our understanding of antibody titre as a correlate of protection, since vaccine effectiveness is usually higher for A(H1N1)pdm09 than for A(H3N2), irrespective of repeated vaccination effects [4]. We could not corroborate these titres values with vaccine effectiveness in our study because there were no infections in 2020 or 2021. However, the threshold for seropositivity may differ for the two influenza A subtypes. Indeed, in a longitudinal household cohort study in Vietnam, the protective titre for A(H1N1)pdm09 was around half the threshold for A(H3N2) [30]. Studies which have combined immunogenicity and vaccine effectiveness data from the same cohort have suggested that the proportion of the vaccine’s effect that is mediated by antibody titre may be low [31], indicating the need for alternative or additional correlates of protection. Measuring the breadth of protection, such as with antibody landscapes that measure responses against several antigens [11], may provide a more robust measure of seroprotection.

In all measures of antibody response explored values for egg-grown antigens were higher than the corresponding cell-grown antigens, even among vaccine-naïve HCWs. This may not be surprising given that all HCWs received egg-based vaccines. We assessed both cell- and egg-grown antigens since the egg-grown antigens can acquire changes that affect immunogenicity and effectiveness [23, 32], while cell-grown antigens may better represent circulating viruses. Strong responses to egg-grown antigens may not provide a reliable correlate of protection against circulating viruses and we therefore recommend caution be applied when interpreting immunogenicity studies that report seropositivity against egg-grown antigens, only.

Our study was hampered by its sample size, particularly for the vaccine-naïve group in 2021 when just 15vaccine-naive HCW were recruited, despite targeted recruitment efforts. The 2021 vaccine-naïve participants were younger and more of them were male than in 2020, which could have contributed to conflicting observations about their antibody response between years. Model fit and precision were also poorer in 2021, despite the overall greater availability of data in that year. It is possible that some of this imprecision results from an actual increase in variability of antibody responses to the new vaccine antigens received compared with 2020. Unfortunately, we had limited opportunity to explore sources of this heterogeneity and whether some of it may have arisen through effect modification.

In conclusion, we observed diminishing antibody responses with successive years of vaccination that was modified by the pre-vaccination titre and to some extent age and vaccine brand. Further analyses on a subset of these HCWs are underway to understand the relative stimulation of *de novo* and recalled B cells that underlie these observations. Expanded cohorts are needed to better understand how attenuated immunogenicity among repeat vaccinees translates to vaccine effectiveness.

## Figures and Tables

**Table 1.** Characteristics of the cohort at baseline visit. Figures are n (%) unless otherwise indicated. Participants without a day 14 blood draw were excluded.

	2020	2021
	n (%)	n (%)
Total	595	1031
Age in years, median (range)	39 (31, 48)	40 (32, 50)
Sex		
Female	483 (81%)	874 (85%)
Male	110 (19%)	155 (15%)
Other	1 (0.17%)	2 (0.19%)
Missing	1 (0.17%)	0 (0%)
BMI, median (range)	24 (22, 28)	25 (22, 29)
≤20	46 (7.7%)	70 (6.8%)
20 - <25	298 (50%)	438 (43%)
25 - <30	158 (27%)	305 (30%)
30 - <35	58 (9.8%)	140 (14%)
35+	34 (5.7%)	73 (7.1%)
Number of years employed, median (range)	4 (2, 9)	5 (2, 12)
Employment status		
Full time	338 (57%)	640 (62%)
Part time	206 (35%)	346 (34%)
Casual	47 (8%)	43 (4.2%)
Missing	4 (0.67%)	2 (0.19%)
Occupation		
Clinical	315 (53%)	473 (46%)
Laboratory	30 (5.1%)	55 (5.3%)
Administrative	75 (13%)	137 (13%)
Other	171 (29%)	364 (35%)
Missing	4 (0.67%)	2 (0.19%)
Work area		
Emergency Department	21 (3.6%)	31 (3%)
Critical Care or Intensive Care U	22 (3.7%)	29 (2.8%)
General Medicine and/or Medical Specialities	26 (4.4%)	36 (3.5%)
Paediatrics and/or Paediatric Specialities	48 (8.1%)	68 (6.6%)
Surgery and/or Surgical Specialties	45 (7.6%)	57 (5.5%)
Gynaecology and/or Obstetrics	6 (1%)	21 (2%)
Oncology and/or Haematology	18 (3%)	23 (2.2%)
Radiology	4 (0.68%)	16 (1.6%)
Outpatient clinic	24 (4.1%)	24 (2.3%)
Pharmacy	5 (0.85%)	12 (1.2%)
Laboratory	25 (4.2%)	54 (5.2%)
Nutrition	2 (0.34%)	2 (0.19%)
Social Work	2 (0.34%)	2 (0.19%)
Physiotherapy	3 (0.51%)	5 (0.49%)
Occupational therapy	1 (0.17%)	4 (0.39%)
Other	185 (31%)	297 (29%)
Multiple	154 (26%)	348 (34%)
Missing	4 (0.67%)	2 (0.19%)
Health conditions		
Cardiac disease	7 (1.2%)	12 (1.2%)
Renal disease	1 (0.17%)	2 (0.19%)
Chronic respiratory condition	26 (4.4%)	49 (4.8%)
Haematological disorder	6 (1%)	7 (0.68%)
Chronic neurological condition	2 (0.34%)	7 (0.68%)
Immunocompromising condition	1 (0.17%)	7 (0.68%)
Diabetes or other metabolic disorder	11 (1.8%)	16 (1.6%)
Smoker	17 (2.9%)	24 (2.3%)
Pregnancy	6 (1%)	16 (1.6%)
Multiple	3 (0.5%)	12 (1.2%)
At least 1 condition	80 (13%)	152 (15%)
Vaccine manufacturer		
GSK	219 (37%)	4 (0.39%)
Sanofi	210 (35%)	885 (87%)
Seqirus	165 (28%)	131 (13%)
Missing	1 (0.17%)	11 (1.1%)
Prior vaccinations		
0	52 (8.7%)	15 (1.5%)
1	71 (12%)	55 (5.3%)
2	69 (12%)	81 (7.9%)
3	67 (11%)	116 (11%)
4	75 (13%)	136 (13%)
5	261 (44%)	628 (61%)

**Table 2.** Incremental effect of years of prior vaccination on day 14 post-vaccination geometric mean titres

Year	Subtype	Substrate	Intercept^*†^	Estimate for linear prior vaccination*	p-value Z-test*	p-value Jonckheere-Terpstra
2020	A(H1N1)pdm09	cell	93 (77, 110)	0.83 (0.79, 0.87)	<0.001	<0.001
		egg	200 (170, 240)	0.85 (0.81, 0.89)	<0.001	<0.001
	A(H3N2)	cell	57 (50, 66)	0.93 (0.9, 0.97)	<0.001	<0.001
		egg	420 (340,510)	0.9 (0.86, 0.95)	<0.001	<0.001
2021	A(H1N1)pdm09	cell	140 (110, 170)	0.79 (0.76, 0.84)	<0.001	<0.001
		egg	500 (400,640)	0.85 (0.8, 0.89)	<0.001	<0.001
	A(H3N2)	cell	94 (77,120)	0.87 (0.83, 0.91)	<0.001	<0.001
		egg	290 (240,350)	0.88 (0.84, 0.92)	<0.001	<0.001

* intercept, Estimate for linear prior vaccination, and p-value (z-test) were produced by fitting a log-linear model to the data with prior vaccination as the only covariate. The effect of prior vaccination is assumed to be linear.

† Intercept is the estimated post-vaccination GMT for the group with 0 prior vaccinations. Estimate for linear prior vaccination is the predicted fold-change for each vaccination (e.g., for 2020 H1 the expected GMT for 2 prior vaccinations is 92 * 0.83 *0.83 = 63). The Jonckheere-Terpstra test is an alternative test for linear trend. P-values assess statistical significance at the α=0.05 level.
